# Identification of Vibration Events in Rotating Blades Using a Fiber Optical Tip Timing Sensor

**DOI:** 10.3390/s19071482

**Published:** 2019-03-27

**Authors:** Dechao Ye, Fajie Duan, Jiajia Jiang, Guangyue Niu, Zhibo Liu, Fangyi Li

**Affiliations:** State Key Laboratory of Precision Measuring Technology and Instruments, Tianjin University, Tianjin 300072, China; fjduan@tju.edu.cn (F.D.); jiajiajiang@tju.edu.cn (J.J.); niuguangyue@tju.edu.cn (G.N.); liu_zhibo1990@163.com (Z.L.); lifangyi0424@163.com (F.L.)

**Keywords:** blade tip timing, blade vibration measurement, identification of vibration events, Pearson correlation coefficient, fiber optical tip timing sensor

## Abstract

The blade tip timing (BTT) technique has been widely used in rotation machinery for non-contact blade vibration measurements. As BTT data is under-sampled, it requires complicated algorithms to reconstruct vibration parameters. Before reconstructing the vibration parameters, the right data segment should first be extracted from the massive volumes of BTT data that include noise from blade vibration events. This step requires manual intervention, is highly dependent on the skill of the operator, and has also made it difficult to automate BTT technique applications. This article proposes an included angle distribution (IAD) correlation method between adjacent revolutions to identify blade vibration events automatically in real time. All included angles of the rotor between any two adjacent blades were accurately detected by only one fiber optical tip timing sensor. Three formulas for calculating IAD correlation were then proposed to identify three types of blade vibration events: the blades’ overall vibrations, vibration of the adjacent two blades, and vibration of a specific blade. Further, the IAD correlation method was optimized in the calculating process to reduce computation load when identifying every blade’s vibration events. The presented IAD correlation method could be used for embedded, real-time, and automatic processing applications. Experimental results showed that the proposed method could identify all vibration events in rotating blades, even small events which may be wrongly identified by skillful operators.

## 1. Introduction

It is important to measure the vibration amplitude of rotating blades in rotational machinery in real-time, which reflects the stress induced in the blades. Dynamic stress is crucial to assess machinery operation state and to predict blade failures. The blade tip timing (BTT) technique, using non-intrusive probes mounted on the engine casing to sense the “arrival time” of passing blades, has become one of the most widely used methods for non-contact blade vibration measurements [1,2,3,4,5]. The BTT technique has advantages over conventional strain gauge stress measuring methods, such as its non-intrusive nature and capability for being used for long-term monitoring. However, the main drawback of the BTT technique is under-sampling, which leads to frequency aliasing [6]. Different BTT data analysis methods for reconstructing vibration parameters have been provided over the past decades [7,8,9,10,11,12,13,14,15]. For synchronous blade vibrations, the least squares sine fitting method is described to predict resonance amplitude and frequency. The fast Fourier transform is often applied to analyze asynchronous blade vibrations. However, the identification of the occurrence of blade vibration events should be carried out before reconstructing vibration parameters, especially for long-term, real-time, and automatic processing applications. An effective method for the identification of blade vibration events without manual intervention will help the BTT technique gain broader applications in industrial automation.

Current approaches to identify blade vibration events from BTT data are threshold value triggering or visual observation by skilled personnel. However, these may produce false identifications due to noise signals or DC drift of data. They also may overlook vibration events which are too small to observe. Rolls-Royce plc suggested an identification method in 2009 to calculate the correlation of the displacement of different probes between current and previous revolutions. Different tip timing probes observe the same “stack pattern” when there is no vibration event, while the “stack pattern” begins to spread as a vibration event commences [16]. For data applied in the calculation of the correlation from different probes, generally four or more, the identification will be more accurate with more probes. However, the inconsistency of different probes could possibly affect the effectiveness of identification. Besides this, the number of probes is strictly limited in actual applications, especially considering the possible failure of probes in long-term monitoring applications.

This article proposes an included angle distribution (IAD) correlation method to identify blade vibration events in actual applications using data from only one fiber optical tip timing sensor. It could be very useful in cases with only one probe or not enough probes for long-term service. Inconsistencies of different probes could not be taken into consideration. The IAD correlation method was used to calculate the included angle distribution’s correlation between adjacent revolutions to identify blade vibration events automatically. Three improved formulas for calculating IAD correlation were suggested to identify three types of blade vibration events: the blades’ overall vibrations, vibration of the adjacent two blades, and vibration of a specific blade. This article also focuses on the aspect of computation load of the method when identifying every blade’s vibration events. The calculating process was optimized to make it suitable for embedded, real-time, and automatic processing applications. Once the vibration events were identified automatically, the following analysis of blade vibration amplitude and frequency through model fitting could be conducted in real time without manual intervention.

## 2. Methodology

### 2.1. BTT System Using Fiber Optical Tip Timing Sensor

**Figure 1:**
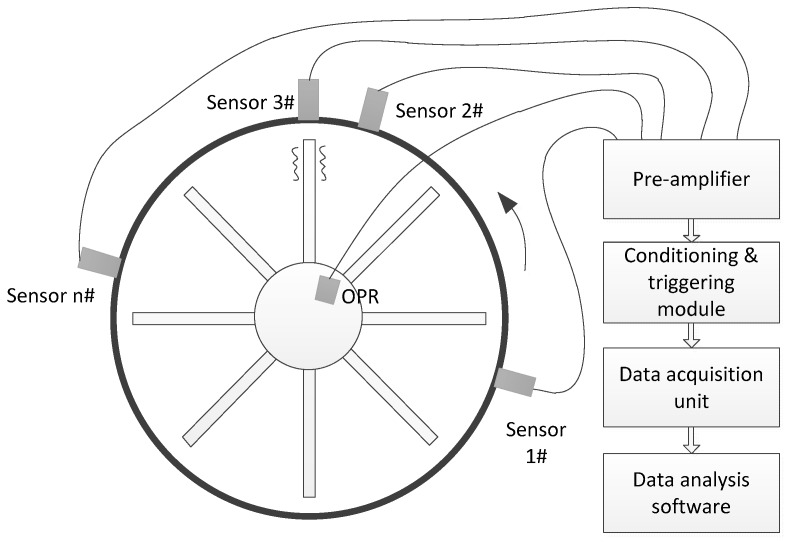
The block diagram of a blade tip timing (BTT) system.

**Figure 2:**
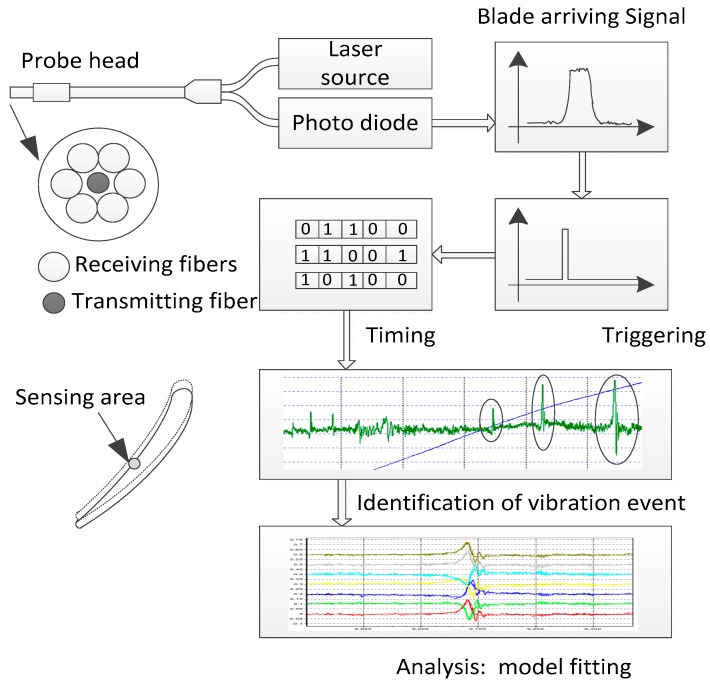
Signal processing of a BTT system.

Typical BTT system for non-contact blade vibration measurement consists of five parts: tip timing sensors including a once-per-revolution (OPR) sensor, pre-amplifier, signal conditioning and triggering module, data acquisition unit, and data analysis software (Figure 1). In general, the tip timing sensor can use fiber optical, capacitance, microwave, or other types of sensors. The sensor senses the arriving signal of passing blades and the preamplifier amplifies and converts it into a voltage output signal. The voltage signal is conditioned and turned into a pulse at the moment of the “arrival time” in the conditioning and triggering module. The arrival time of passing blades can then be acquired by timing the rising edge of the pulse, as shown in Figure 2.

However, the BTT data obtained from the data acquisition unit is under-sampled. The sample rate is equal to the rotor rotational speed. It is necessary to reconstruct the vibrations’ parameters using model fitting algorithms, which were carried out in the data analysis software. Many model fitting algorithms to reconstruct blade vibration information have been well established, though this paper will not discuss those algorithms. However, those algorithms require appropriate and correct input data—that is, they need to complete the identification of vibration events before model fitting. This paper proposes the use of one tip timing sensor, in particular a fiber optical tip timing sensor, to identify blade vibration events in a BTT system. The fiber optical sensor has advantages of higher resolution, lower noise level, and faster response capability. It can sense smaller blade tip vibration displacements than other types of sensors. The identification of blade events could be carried out in a computer-based software or data acquisition unit for embedded, real-time, and automatic processing applications.

The optical fiber tip timing sensor used in this paper consists of a transmitting fiber in the center of the probe head and six receiving fibers around, as shown in Figure 2. The probe head is mounted on the engine casing and transmits a beam of light from the laser source, sensing the reflected light when the blade passes through. The fiber optical tip timing sensor’s sensing area on a blade is related to the core diameter and numerical aperture (NA) of the transmitting optical fiber. When the core diameter is 62.5 μm and NA = 0.12, the diameter of the sensing area is smaller than 1.20 mm, meaning that the sensing resolution is much better than eddy current, capacitive, or other types of probes [17,18,19].

### 2.2. IAD Correlation Method to Identify Blade Vibration Events

**Figure 3:**
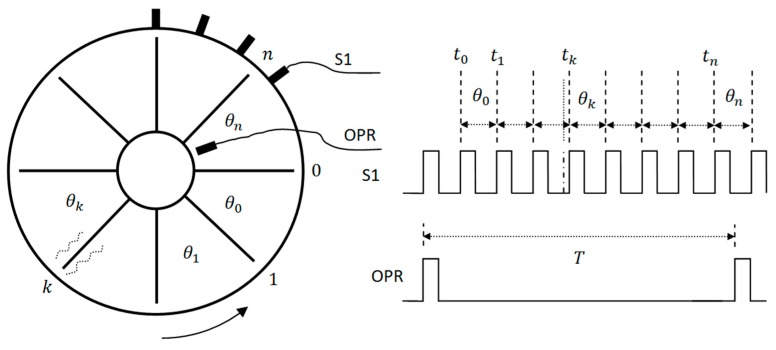
Measurement of the included angle distribution.

As shown in Section 2.1, tip timing sensors can sense the arrival time of all blades. The small changes of included angles between any two adjacent blades can be also sensed using one fiber optical tip timing sensor. The sensor obtains the arrival time ($t_{k}$) of all blades and the OPR sensor obtains the rotation period (T) (Figure 3). The included angle ($\theta_{k}$) between blade *k#* and *k*+1# of the rotor can then be obtained from Equation (1). In a BTT system, the included angle between two adjacent blades can be measured at the same time. So, the IAD correlation method was proposed to identify blade vibration events:

(1) $$\theta_{k}=\frac{2\pi [t_{k+1}-t_{k}]}{T}.$$

It is assumed that the rotor’s included angle distribution of the previous revolution is $\phi_{p}=(\theta_{0p},\theta_{1p},\cdots ,\theta_{kp},\cdots ,\theta_{np})$, where $\theta_{kp}$ refers to the included angle between blade *k#* and *k*+1# of the previous revolution and *n* is the last number of blades. Besides this, $\phi_{c}=(\theta_{0c},\theta_{1c},\cdots ,\theta_{kc},\cdots ,\theta_{nc})$ refers to the included angle distribution of the current revolution. When any blade is vibrating, $\phi_{c}$ will change and be different from $\phi_{p}$.

The IAD correlation method calculates the Pearson correlation coefficient between included angle distributions of previous and current revolutions ($r_{pc}$) to identify blade vibration events, as seen in Equation (2). In Equation (2), $\bar{\theta_{p}}$ and $\bar{\theta_{c}}$ are the average values of $\phi_{p}$ and $\phi_{c}$, respectively.

(2) $$r_{pc}=corr(\phi_{p},\phi_{c})=\frac{\sum_{i=0}^{n}\left[\theta_{ip}-\bar{\theta_{p}}][\theta_{ic}-\bar{\theta_{c}}\right]}{\sqrt{\sum_{i=0}^{n}{\left[\theta_{ip}-\bar{\theta_{p}}\right]}^{2}}\sqrt{\sum_{i=0}^{n}{\left[\theta_{ic}-\bar{\theta_{c}}\right]}^{2}}}$$

This could be improved to amplify the changes of the $r_{pc}$ value, where $a_{1}$ is a scaling factor, as seen in Equation (3). In a normal non-vibrating state, $r_{pc}$ is equal to 1. The value of $r_{pc}$ will decrease while some blades are vibrating.

(3) $$r_{pc}=1-(1-corr(\phi_{p},\phi_{c}))\times a_{1}$$

Changes in the rotor’s working environment, such as changes of airflow or rotational speed, may also lead to a variation of $r_{pc}$. These variations are much slower than those caused by blade vibrations. If $\phi_{p}$ is obtained on the adjacent revolution or adjacent several revolutions before the current revolution, the variation of $r_{pc}$ is small due to the changes in the rotor’s working environment. As the change of the included angle between the adjacent revolution or adjacent several revolutions is very small, the tip timing sensor requires high resolution capability. A fiber optical tip timing sensor meets this requirement. Equation (3) shows that the rotor is suffering blade vibrations when $r_{pc}<Th_{1}$. $Th_{1}$ is the threshold value. As the vibrations of any blade or several blades would lead to the decrement of the $r_{pc}$ value, Equation (3) can be used to identify the rotor’s overall vibration event.

However, Equation (3) cannot be used to identify which blades are vibrating. As the vibration of blade *k#* and *k*+1# will both affect the value of $\theta_{kc}$, an improved calculating formula of IAD correlation method to identify vibration events of the adjacent two blades is proposed in Equation (4). Assuming $\phi_{c}^{k}=(\theta_{0p},\theta_{1p},\cdots ,\theta_{kc},\cdots ,\theta_{np})$, $r_{pc}^{k}$ will be equal to 1 in a normal non-vibrating state. $a_{2}$ is a scaling factor.

(4) $$r_{pc}^{k}=1-(1-corr(\phi_{p},\phi_{c}^{k}))\times a_{2}$$

Assuming $Th_{2}$ and $Th_{3}$ are threshold values, if $r_{pc}^{k}<Th_{2}$, then blades k# and *k*+1# may be vibrating. Equation (5) is a further improved formula for calculating IAD correlation to identify the vibration events of the specific blade *k#*. Equation (5) could eliminate the effect of blade *k*+1# because the vibration of blade *k*+1# would not affect the $r_{pc}^{k-1}$ value. The vibration of blade *k*# can lead to decrements of $r_{pc}^{k}$ and $r_{pc}^{k-1}$ at same time. This indicates that blade *k*# is vibrating when $i^{k}$ >$Th_{3}$. $a_{3}$ is a scaling factor.

(5) $$i^{k}=\left\{ \begin{array}{l} \left(1-r_{pc}^{k})\times (1-r_{pc}^{k-1})\times a_{3},if(r_{pc}^{k}<Th_{2}\right)\\ 0,if(r_{pc}^{k}\geq Th_{2}) \end{array}\right.$$

A practical method to determine the values of $Th_{1}$, $Th_{2}$, and $Th_{3}$ is proposed in the following steps:

1. Calculate the values of $r_{pc}$, $r_{pc}^{k}$, and $i^{k}$ during a period of time when there is no vibration event in the rotating blades, and find their values when the blade deflection’s noise achieves its maximum. Record the values of $\phi_{p}$ and $\phi_{c}$ simultaneously.
2. According to the signal to noise ratio of BTT data and the radius of the blade tip, set the minimum vibration amplitude to be detected, and then calculate the changes of the included angle between the adjacent two blades caused by that vibration. Refresh the values of $\phi_{c}$. It is noteworthy that if the minimum vibration amplitude is set too small, a blade event may be incorrectly identified.
3. Recalculate the values of $r_{pc}$, $r_{pc}^{k}$, and $i^{k}$ with the updated values of $\phi_{c}$, which can be used as the values of $Th_{1}$, $Th_{2}$, and $Th_{3}$ respectively.

It is noteworthy that in Equation (5), if blade *k*−1# and *k*+1# are both vibrating but at the same time blade *k*# does not have any vibration, the $i^{k}$ value will increase. In this case, a incorrect identification of a blade event may occur, though the probability of this occurrence is relatively low in practical applications. Other factors could be taken into consideration to assist in identifying vibration events of blade *k*#, such as amplitude threshold triggering.

### 2.3. Optimized Calculation Process

In order to be used in the health monitoring of industrial machinery the IAD correlation method should have the capability of fast and real-time processing. The calculating process of the IAD correlation method could be expanded into an iterative procedure with less computation. From the definition of the Pearson correlation coefficient formula, five components ($\sum x_{i}$, ${\sum x_{i}}^{2}$, $\sum x_{i}y_{i}$, $\sum y_{i}$, and ${\sum y_{i}}^{2}$) are involved in the calculation, shown as Equation (6). Assuming that $H_{1}$, $H_{2}$, $H_{3}$, $H_{4}$, and $H_{5}$ refer to $\sum x_{i}$, ${\sum x_{i}}^{2}$, $\sum x_{i}y_{i}$, $\sum y_{i}$, and ${\sum y_{i}}^{2}$ of blade **0#**, respectively, only three components need additional calculation when identifying vibration events of blade *k*#. Thus, the calculation of blade k# using Equation (4) or Equation (5) could be derived from blade 0#, shown as Table 1.

(6) $$corr(x,y)=\frac{N\sum x_{i}y_{i}-\sum x_{i}\sum y_{i}}{\sqrt{N\sum x_{i}{}^{2}-{\left(\sum x_{i}\right)}^{2}}\sqrt{N\sum y_{i}{}^{2}-{\left(\sum y_{i}\right)}^{2}}}$$

The optimized calculation process means that the IAD correlation method proposed in this paper is suitable for identifying vibration events of every blade with less computation, meeting the requirements of real-time, embedded, and automatic processing applications.

## 3. Experimental Results and Discussion

### 3.1. High-Speed Bench Test

**Figure 4:**
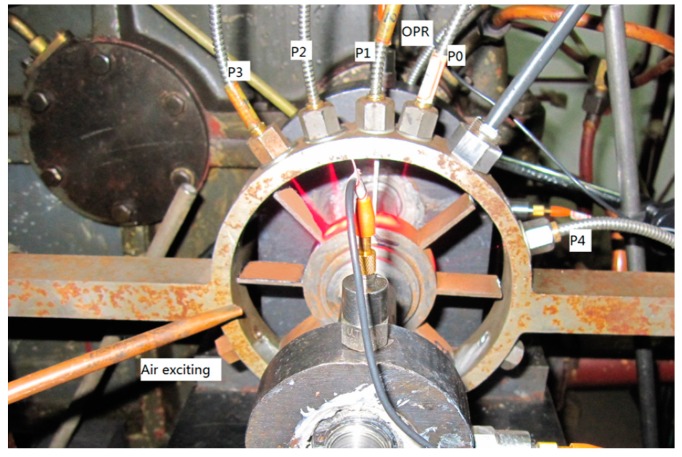
Test rig and installation of fiber optical tip timing sensors.

In order to verify the effectiveness of the IAD correlation method, five fiber optical sensors numbered as P0 to P4 were mounted on a high-speed test rig for a blade vibration measuring experiment. The installation of the sensors is shown in Figure 4. The rotor has a total of eight blades, and the radius of each blade tip is 60 mm. The thickness of the blades is 2 mm. Blades were excited by nitrogen with high pressure during the experiment. Synchronous vibration will occur when blade natural frequency is an integral multiple of rotational frequency. This integral number is often called the engine order (EO). Besides this, the blade will also be bended slightly due to air force.

**Figure 5:**
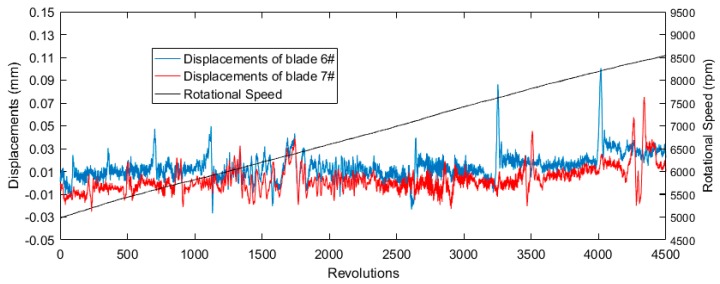
Blade vibration deflection and rotation speed waveforms.

In this paper, the data of the P0 sensor is used to identify blade vibration events. The displacements of blade 6#, blade 7#, and the rotational speed ranging from 5000 rpm to 8500 rpm are shown in Figure 5. Both blades experienced several first-order synchronous vibrations under the excitation of nitrogen. The two blades have different vibration amplitudes and natural frequencies with noises in the waveforms. Thus, it is likely certain vibration events will be misjudged using methods of threshold value triggering or visual observation by skilled personnel.

### 3.2. Identification of Blades’ Overall Vibration Events

**Figure 6:**
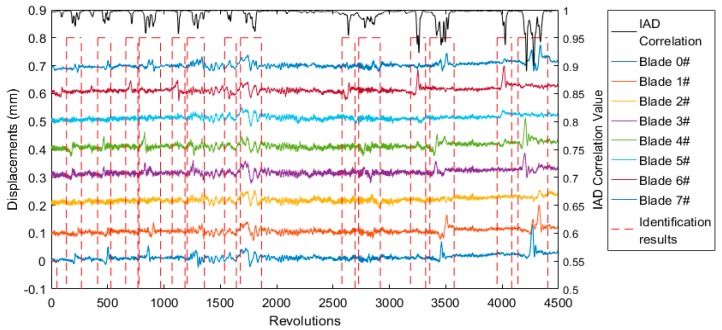
Identification results of blades’ overall vibration events using Equation (3).

As shown in Equation (3), the overall vibration events of blades during the test can be identified, and the results are shown in Figure 6. All eight blades experienced first-order bending vibrations due to the excitation of nitrogen, when blade natural frequency is an integral multiple of rotational frequency. The displacements of all eight blades are also shown in Figure 6, separated 0.1 mm apart from each other. The displacements varied when the blades were experiencing vibration events, and the vibration amplitudes were different from each other. Setting $Th_{1}$ = 0.98 indicates the occurrence of a blade vibration event if $r_{pc}$ < $Th_{1}$. The identification results were then set at a high level, including the previous 50 revolutions and subsequent 50 revolutions, providing adequate data for model fitting. In this paper, the identification results were all set as dashed lines to highlight the vibration events in the displacement waveforms.

As shown in Figure 6, the overall vibration events of blades were correctly identified. The vibrations of any blade or several blades led to a decrement of the $r_{pc}$ value. The variation of displacements at around 1750 revolutions may not truly be a blade vibration event, which can be eliminated using Equation (4) or Equation (5). In addition, some tiny blade vibration events ranging from 2000 to 2500 revolutions were not identified.

### 3.3. Identification of Adjacent Two Blades’ and a Specific Blade’s Events

**Figure 7:**
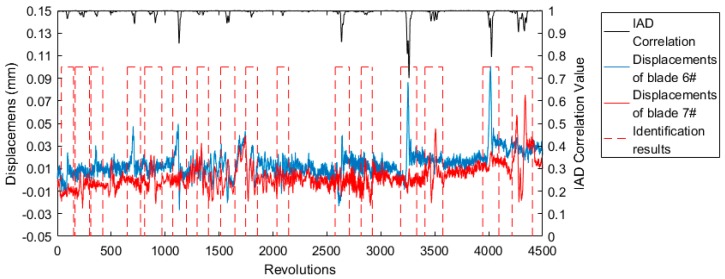
Identification results of blade 6# and 7# using Equation (4).

According to Equation (4), the identification results of adjacent two blades’ events are shown in Figure 7, where $Th_{2}$ was set equal to 0.98. The $r_{pc}^{6}$ value decreased significantly when blade 6# or blade 7# was vibrating. The tiny blade vibration events between 2000 and 2500 revolutions were successfully identified.

**Figure 8:**
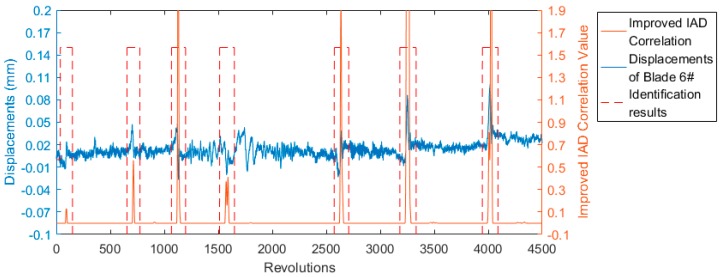
Identification results of blade 6# using Equation (5).

Equation (5) was used to identify a specific blade’s vibration events. The identification results of blade 6# are shown in Figure 8, where $Th_{3}$ = 0.04. Figure 8 indicates that the IAD correlation method using Equation (5) can accurately identify all of the vibration events and eliminate the interference of adjacent blades. Even skilled personnel may make mistakes when trying to identify signals between 1500 and 2500 revolutions. The large signal around 1700 revolutions was not actually a real vibration event. It might have been caused by the rotating shaft or the OPR sensor, which cannot affect the included angle distribution of rotor. The method proposed in this paper can successfully eliminate such interferences.

**Figure 9:**
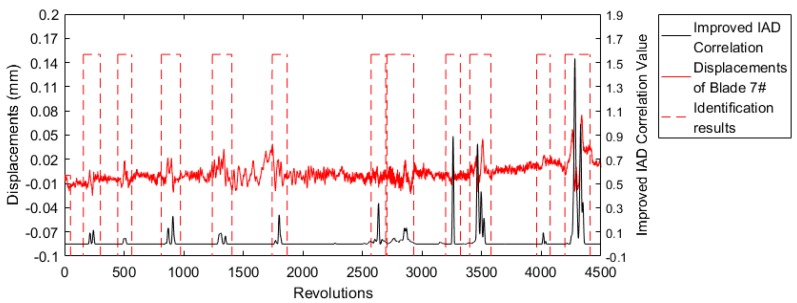
Identification results of blade 7# using Equation (5).

Figure 9 shows the identification results of blade 7# using Equation (5). The vibration events of just blade 7# itself were identified as well as the coupled vibration events at 2600, 3300, and 4000 revolutions. These coupled vibration events were introduced by the large vibration of the adjacent blade 6#. Such tiny coupled vibration signals could also be identified using Equation (5).

**Figure 10:**
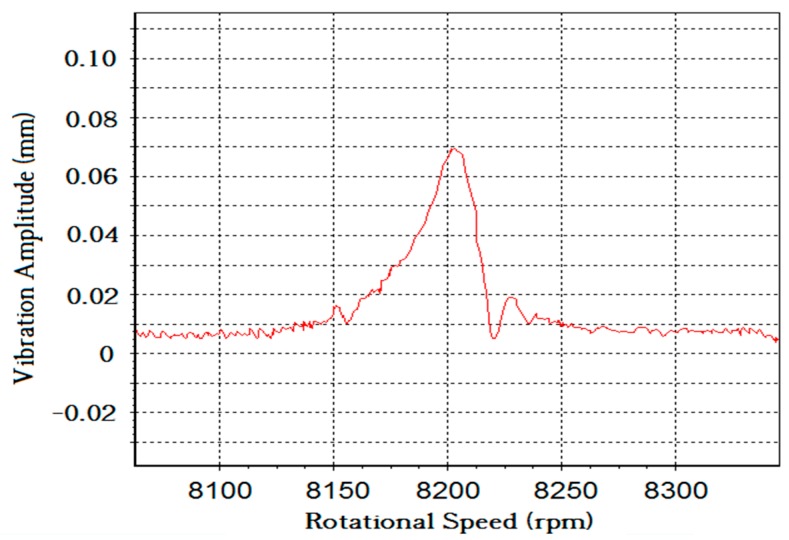
Model fitting result of blade 6# at 8200 rpm, engine order (EO) = 13.

Once the blade vibration events were correctly identified in real time, BTT data analysis could be conducted to reconstruct the vibration amplitude and frequency. In this paper, a least squares fitting method was used to model the fit of the BTT data. The model fitting result of blade 6# around 4000 revolutions is shown in Figure 10. The amplitude reached a maximum of 0.07 mm at 8201 rpm, the engine order (EO) was 13, and the first-order vibration frequency was 1777.02 Hz. All other vibration events of Figure 8 were extracted to carry out BTT data analysis using the least squares fitting method. The results are shown in Table 2.

**Figure 11:**
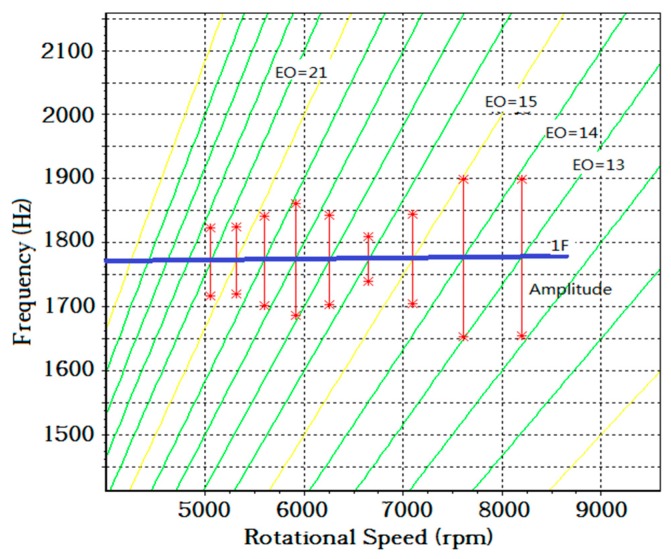
Campbell diagram of blade 6#.

The model fitting results are listed in the Campbell diagram (Figure 11). Figure 11 could also prove that the vibration events of blade 6# were correct. Synchronous vibration occurred when, theoretically, the blade natural frequency is an integral multiple of rotational frequency. The results in Figure 11 accurately fell on all of the intersect points between the first-order vibration wave line and EO lines.

### 3.4. Comparison of the IAD Correlation Method with the Probe Displacement Distribution (PDD) Correlation Method

**Figure 12:**
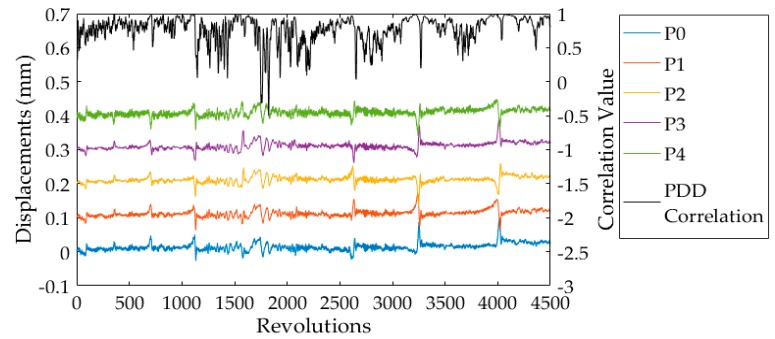
Identification results of blade 6# using the probe displacement distribution (PDD) correlation method.

In Reference [16], it was suggested that the displacement value of a single blade at each probe is correlated to the values for the next rotation. This reference then proposed a method to identify blade vibration events by calculating Pearson correlation coefficient between the probe displacement distributions of previous and current revolutions (also called PDD correlation method in this paper). The identification results of blade 6# based on the PDD correlation method are shown in Figure 12. The displacements of blade 6# from five probes are also shown in Figure 12, which were separated 0.1 mm apart from each other. Compared to the IAD correlation method, it is difficult to set a threshold value to indicate the occurrences of vibration events according to the results of the PDD correlation method. As the data applied in that calculation of correlation were from five different probes, the inconsistency of different probes, including the noises in displacement signals, affected the correlation value.

## 4. Conclusions

In this paper, an IAD correlation method to identify vibration events automatically for rotational blades of machinery was proposed. All included rotor angles between any two adjacent blades were accurately detected by only one fiber optical tip timing sensor, and then three formulas for calculating the IAD correlation were proposed to identify three types of blades vibration events: the blades’ overall vibrations, vibration of the adjacent two blades, and vibration of a specific blade. Further, the IAD correlation method was optimized in the calculating process to reduce computation load while identifying every blade’s vibration events. The method can meet the requirements of real-time, embedded, and automatic processing applications.

The experimental results showed that the proposed method could effectively identify all vibration events. Compared with the visual observation method or the simple threshold value triggering method, the present method eliminated fake vibration signals, which may be wrongly recognized as vibration events by human judgment. The coupled vibration signals of adjacent blades were also accurately identified. The three calculating formulas described in this paper provide objective criterions for the identification of the blades’ overall vibration, vibration of adjacent two blades, and vibration events of a specific blade, and are suitable for different applications.

Although synchronous vibration events in the experiments were only air-excited, other types of blade vibration events will also lead to changes in the included angle distribution of rotor blades, such as foreign object damage (FOD) events and blade asynchronous vibration events. Therefore, the IAD correlation method has general applicability to be used for the identification of such types of events. The response of the blade to FOD events is complex. There is no effective method to identify FOD events of blades at present. This can be further studied in combination with the technology described in this paper.

## Figures and Tables

**Table 1:** Optimized calculation of $r_{pc}^{k}$.

$r_{pc}^{k}$	$\sum x_{i}$	${\sum x_{i}}^{2}$	$\sum x_{i}y_{i}$	$\sum y_{i}$	${\sum y_{i}}^{2}$
$r_{pc}^{0}$	$H_{1}$	$H_{2}$	$H_{3}$	$H_{4}$	$H_{5}$
$r_{pc}^{1}$	$H_{1}$	$H_{2}$	$H_{3}$+$\theta_{0p}$($\theta_{0p}$−$\theta_{0c}$)−$\theta_{1p}$($\theta_{1p}$−$\theta_{1c}$)	$H_{4}$+ ($\theta_{0p}$−$\theta_{0c}$)−($\theta_{1p}$−$\theta_{1c}$)	$H_{5}$+($\theta_{0p}$−$\theta_{0c}$)($\theta_{0p}$+$\theta_{0c}$)−($\theta_{1p}$−$\theta_{1c}$)($\theta_{1p}$+$\theta_{1c}$)
…	…	…	…	…	…
$r_{pc}^{k}$	$H_{1}$	$H_{2}$	$H_{3}$+$\theta_{0p}$($\theta_{0p}$−$\theta_{0c}$)−$\theta_{kp}$($\theta_{kp}$−$\theta_{kc}$)	$H_{4}$+ ($\theta_{0p}$−$\theta_{0c}$)−($\theta_{kp}$−$\theta_{kc}$)	$H_{5}$+($\theta_{0p}$−$\theta_{0c}$)($\theta_{0p}$+$\theta_{0c}$)−($\theta_{kp}$−$\theta_{kc}$)($\theta_{kp}$+$\theta_{kc}$)
…	…	…	…	…	…

**Table 2:** Model fitting results of blade 6#.

Engine Order	Amplitude (mm)	Frequency (Hz)	Center Speed (rpm)
13	0.07	1777.02	8201.61
14	0.07	1776.07	7611.72
15	0.04	1775.02	7100.07
16	0.02	1774.22	6653.32
17	0.04	1773.28	6258.62
18	0.05	1773.38	5911.26
19	0.04	1772.00	5595.79
20	0.03	1772.59	5317.77
21	0.03	1770.06	5057.30
